# Genetic polymorphism of Pro12Ala in type 2 diabetes mellitus: Role in inflammation linked to Insulin resistance

**DOI:** 10.6026/97320630019946

**Published:** 2023-09-30

**Authors:** Venkata Ramesh Bonam, Abu Raghavan Srinivasan, Manoj DanielDevaprasad, Ranjith Babu Kudikala

**Affiliations:** 1 Department of Biochemistry, Apollo Institute of Medical Sciences and Research (AIMSR), The Apollo University, Chittoor campus, Murukambattu, Chittoor -517127, Andhra Pradesh, India; 2 Department of Biochemistry, Mahatma Gandhi Medical College and Research Institute, Sri Balaji Vidyapeeth (SBV), SBV Campus, Pillaiyarkuppam, Pondicherry - 607 402, India; 3 Department of General Medicine, Apollo Institute of Medical Sciences and Research (AIMSR), The Apollo University, Chittoor campus, Murukambattu, Chittoor -517127, Andhra Pradesh, India; 4 Department of Physiology, Government Medical College, Rajanna Sircilla, Telangana - 505 301, India.

**Keywords:** Genetic polymorphism, Pro12Ala, type 2 diabetes mellitus, inflammation, Insulin resistance

## Abstract

Peroxisome Proliferator-Activated Receptor gamma 2 (PPARγ2) belongs to nuclear receptor superfamily and plays a role in adipocyte differentiation and inflammation. Evidences suggest that inflammatory processes hold key to insulin resistance and PPARγ2 has also been implicated. PPARγ2 exhibits gene polymorphism. The Ala allele of Pro12Ala polymorphism (rs1801282) is associated with a reduced risk for insulin resistance. We attempted the study in overweight and obese males to generate evidences linking insulin resistance, inflammatory mediators, and gene polymorphism of PPARγ2 in overweight and obese males. The conventional biochemical parameters were estimated using established methods. Adiponectin and Haptoglobin were quantitated by ELISA, whereas Ferritin and hs-CRP were by chemi-luminescence. Indices of insulin sensitivity /Insulin resistance were computed based on established formulae. Gene analysis was based on PCR and RFLP. Appropriate statistical analysis was enabled to project gene polymorphism.The heterozygous variant (CG) was around 8 and 38 percent respectively in overweight and obese males. The G Allele was 3.89% and 18.82%. The wild type and heterozygous variant of PPARγ2 depicted significance with haptoglobin, whereas adiponectin showed significance in the wild type. Chi-square test was performed to assess the relation between polymorphic genotypes and ferritin emerged significant. Indices of insulin resistance showed different characteristics with wild type and heterozygous variant ofPPARγ2 gene polymorphism. The inflammatory mediators (hs-CRP, Ferritin, Haptoglobin and adiponectin) exhibited variegated characteristics with the wild type and heterozygous variant of PPARγ2, thus pointing to the nexus among insulin resistance, inflammation, and adipocyte differentiation.

## Background:

Genetic polymorphisms are widely believed to influence the risk for T2DM and the subsequent progression of insulin resistance. Peroxisome proliferator-activated receptor (PPAR)-gamma is regarded as a transcription factor with a well projected role in adipocyte differentiation [1]. The Ala allele of the common Pro12Ala polymorphism in the isoform PPAR-gamma 2 possesses a nexus with a lessened risk for type 2 diabetes mellitus (T2DM) .In addition to adipocyte differentiation, other events namely lipid, glucose homeostasis, and insulin sensitivity are considered as cardinal cellular events that are influenced by the transcription factor PPARγ. PPARγ is a nuclear hormone receptor and known for its genetic polymorphisms [2].PPARγ hetero-dimerizes with retinoid X receptor (RXR).This further binds to co-repressors and activates target gene expression[3].PPARγ is located in chromosome 3 and typified by >150kb genomic segment. There are two distinct isoforms of PPARγ, namely PPARγ1 and PPARγ2. P1 promoter translates PPARγ1 from its mRNA which comprises exons A1, A2, and 1-6, while the P2 promoter translates PPARγ2 from its mRNA which is a combination of exons B and 1-6. There is a copious expression of PPARγ in adipocytes, macrophages, and the large intestine. However, PPARγ2 expression is limited to adipocytes and hence acquires immediate relevance in insulin resistance and T2DM. We have attempted this study by trying to relate the role of inflammatory mediators, namely ferritin, haptoglobin and hsCRP with reference to PPARγ2 polymorphism, but with reference to obese and overweight males. HbA1c (Glycated hemoglobin) is a gold standard for monitoring glycemic control in patients with T2DM.Poor glycemic control in T2DM patients often leads to a dyslipidemic state. Increased HbA1c is a risk factor for cardiovascular disorders in patients with diabetes. High-risk T2DM individuals are those with metabolic syndrome, as they have a very high risk of cardiovascular complications [4]. T2DM-associated micro and macrovascular complications reveal a close connection between glycemic control and lipid profile. Poor glycemic control is directly proportional to abnormal lipid levels [5]. Several clinical studies have demonstrated the anti-inflammatory properties of adiponectin and its beneficial effects on metabolic and cardiovascular health. Adiponectin elicits its direct actions in the skeletal muscle, liver, and vasculature. In humans, adiponectin replacement therapy is being considered as a better strategy in the treatment of obesity, atherosclerosis, and insulin resistance/type 2 diabetes [6].The need for the present investigation stems from the fact that insulin resistance is synonymous with T2DM. We made a humble attempt to compare and elucidate the insulin resistance in obese and overweight T2DM patients (males) through the assessment of biochemical markers of inflammation and atherogenicity such as ferritin, hs-CRP, Haptoglobin with reference to the gene polymorphism (Pro12 Ala) of peroxisome proliferator-activated receptor gamma (PPAR- γ). Therefore, it is of interest to document the genetic polymorphism of pro12Ala in type 2 diabetes mellitus in the perspective of insulin resistance and mediators of inflammation

##  Study subjects and methods:

## Study design:

The study was carried out in South India, at a health care establishment offering tertiary care. The study comprised of 180 male diabetics (T2DM). Ninety overweight subjects and an equal number of obese subjects, to be precise were the study subjects. The study design received the approval of the Research Advisory Committee (RAC) and Institutional Human Ethics Committee (File no IEC15/AIMSR/02/2018). Based on the anthropometric index, namely BMI, obese and overweight subjects were segregated. Informed consent was obtained from the participants after duly explaining the purpose and benefits of the study in the vernacular language. The diagnosis of T2DM in the study subjects was enabled by qualified clinicians possessing a valid registration in the respective medical council. T2DM was confirmed both clinically and biochemically. Type 2 diabetics with a duration of more than five years were only included in the study. All the study subjects were maintained on standard care (diabetic medication) and as per the institute's rational drug policy. Patients with thyroid and other endocrine abnormalities, organ dysfunction, systemic, and inflammatory diseases were promptly excluded from the study [7].

## Processing of blood samples:

Venous blood samples from the study subjects were drawn into 5 ml EDTA vacutainers for the purpose of enabling biochemical quantitation and genetic polymorphism studies. Furthermore, two mL of the blood samples were stored at -70 °C until the analysis of single nucleotide polymorphism of PPARgamma2 (Pro12 Ala). In addition, three ml of blood was withdrawn for enabling other biochemical parameters. The study subjects were informed in a prior manner about the minor discomfort in providing the samples of blood.

## Measurement of biochemical parameters:

Blood glucose (fasting, post prandial)was estimated based on an established method complying with enzymatic, automated procedure. Glycated hemoglobin (HbA1C), a measure of glycemic control was quantitated by High Performance Liquid Chromatography (HPLC). Quantitative insulin sensitivity check index, abbreviated as QUICKI was computed based on a formula. Insulin resistance was enabled by the surrogate marker HOMA - IR (Fasting plasma glucose (mmol/l) x plasma fasting insulin (m IU/ml)/22.5). HOMA-IR was considered high when the value was ≥ 2.69[134]. HOMA-BETA (β), a measure of the beta cell mass was assessed by employing the formula HOMA-B = 20 x insulin/(glucose - 3.5) [8].

The inflammatory biomarkers that were estimated in the study namely ferritin, haptoglobin, adiponectin, and hs-CRP were enabled by established methods using approved procedures. Adiponectin and Haptoglobin were estimated by ELISA. Ferritin and hs-CRP were quantitated by automated chemiluminescence. Triacylglycerols and total cholesterol were estimated by enzymatic method. HDL cholesterol was quantitated based on polyanion precipitation. LDL cholesterol was calculated using the Friedwald equation, as in our study, the subjects possessed triacylglycerols less than 400 mg/dl. Small dense LDL particle size was quantitated using the surrogate marker (TAG/HDL). The atherogenic index of plasma (AIP) was computed by employing the online calculator and based on TAG and HDL values [9].

## Genetic Polymorphism Studies:

## Isolation of genomic DNA, amplification, and RFLP (Restriction Fragment Length Polymorphism):

Genomic DNA was isolated from whole blood utilizing Qiagen's Fast DNA tissue kit (procedure as per manufacturer's instructions). Samples were homogenized using a mix of mechanical, chemical, and enzymatic lysis in the QIAamp Fast DNA Tissue Kit. The homogenisation was enabled in as short as five minutes on a desktop vortex. Proteinase K was also present in the digestion buffer mix. Following the purification of genomic DNA, it was delivered for amplification with PCR. To determine the genotype frequency of PPAR gamma 2 (Pro12Ala), gene amplification was performed with PCR. Amplified PCR products were then subjected to restriction digestion with Hga I restriction enzyme (RFLP) to quantify the three genotypes, namely wild type (CC), heterozygous type (CG), and homozygous mutant (GG) in the South Indian population. Under optimal conditions, the restriction-digested end products were separated on a 2.5% agarose gel [10].

## Agarose gel electrophoresis:

**Figure 1:**
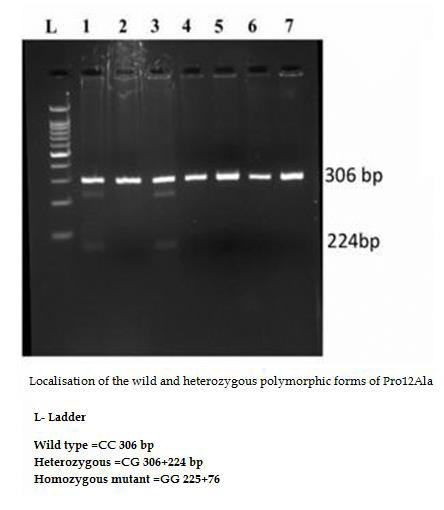
The PPARγ Pro 12 Ala genotype analysis by RFLP-PCR

DNA was duly placed into pre-cast gel wells for enabling agarose gel electrophoresis and an appropriate electric current was applied to resolve the DNA. The DNA so separated was visualised under UV light, following suitable staining [11].The genotype pattern is portrayed in Figure 1.The details are provided in Tables 2 to 4.

## Statistical analysis:

With reference to the allelic distribution of the Single Nucleotide Polymorphism, namely PPAR Gamma 2 (Pro12Ala gene), it was tested for Hardy-Weinberg equilibrium (p < 0.05). Further, the objective proportion of the genotypes pertaining to the alleles was compared through the statistical analysis, namely Pearson χ², odds ratios and 95% confidence intervals (CI). Pearson's correlation matrix was suitably deployed to evaluate the correlation between biochemical parameters and the genotypes. Statistical analysis of the data obtained was duly performed with SPSS & Graph Pad Prism software version 8.0.0 for Windows, USA, www.graphpad.com.

## Results and Discussion:

##  Agarose gel electrophoresis depicting the PCR-RFLP pattern of PPAR gamma 2:

## Distribution of polymorphism-Overweight vs. Obese:

Figure 1shows the PPARγ Pro 12 Ala genotype by RFLP-PCR method. The details of the distribution of polymorphism-Overweight vs. Obese (Genotyping between two groups) were detailed in Table 1. Table 2 includes the assessment of biochemical parameters in obese and overweight T2DM with PPARγ polymorphism.

##  Effect of PPAR gamma 2 polymorphism on glycemic control in obese and overweight T2DM patients:

**Figure 2:**
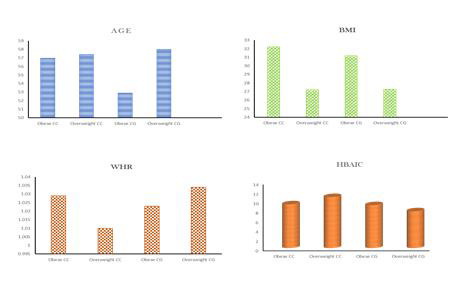
Comparative analysis of age, BMI, WHR and HBA1C with respect to polymorphism type in both obese and overweight

The current data suggests that glycated hemoglobin levels in PPARγ2 wild overweight (10.83± 12.99) T2DM patients are significantly higher when compared to PPARγ2 heterozygous overweight (7.814± 1.276) (Table 2, Figure 2)

## Role of PPAR gamma 2 polymorphism in generating IR in obese and overweight T2DM patients:

**Figure 3:**
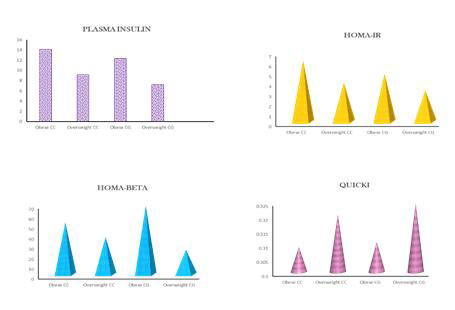
Comparative analysis of Plasma Insulin, HOMA-IR, HOMA-BETA and QUICKI with respect to polymorphism type in both obese and overweight

HOMA-IR calculation reveals that both wild obese T2DM had a 3-fold increase (6.022± 6.607) in insulin resistance when compared to heterozygous obese T2DM with a 1-fold increase (3.087± 0.7427). In the case of overweight T2DM patients, both wild (3.834± 2.768) and heterozygous (3.087± 0.7427) PPARγ2 polymorphic individuals had a 1-fold increase in insulin resistance. This data suggests the susceptibility of the wild PPARγ2 gene to insulin resistance (Table 2, Figure 3).

## Role of PPAR gamma 2 polymorphism in beta cell function (HOMA-BETA):

HOMA-BETA values obtained from the analysis of wild and heterozygous PPARγ 2 polymorphic T2DM patients demonstrate that there is significant β cell dysfunction observed in heterozygous (24.47± 11.98) overweight T2DM individuals when compared to wild (37.27± 44.92) overweight T2DM individuals. To note, changes were not found to a great extent in the β cell dysfunction particularly in obese wild (51.78± 42.03) and heterozygous (68.23± 81.64) PPARγ 2 polymorphic T2DM patients (Table 2, Figure 3). These suggest that heterozygous overweight T2DM have increased β cell dysfunction which could ultimately account for the further progression of insulin resistance.

## hs-CRP in obese and overweight T2DM patients with reference to PPAR gamma 2 polymorphism:

The inflammatory biomarker hs-CRP levels seem to be elevated above the normal levels in both wild and heterozygous PPARγ 2 polymorphic obese and overweight T2DM subjects. A drastic hike in the hs-CRP levels was observed in heterozygous obese (5.481 ± 3.468) T2DM followed by wild (4.584 ± 3.301) overweight T2DM patients (Table 3).

## Influence of PPAR gamma 2 polymorphism over adiponectin in obese and overweight T2DM patients:

Adipose tissue-derived peptide hormone adiponectin was assessed in obese and overweight T2DM patients with PPAR γ 2 polymorphism. The result indicates that there was a significant decrease in adiponectin in both wild and heterozygous polymorphic groups (Table 3). The decrease in the circulating levels of adiponectin observed in the present study can be negatively correlated with insulin resistance.

## Quantitation of Ferritin in obese and overweight T2DM with PPAR gamma 2 polymorphism:

The ferritin levels assessed in wild and heterozygous PPARγ2 polymorphic obese and overweight T2DM subjects indicated no significant increase. The serum ferritin levels were observed to be within the normal range (Table 3).

## PPAR gamma 2 polymorphism in the light of the acute phase protein, haptoglobin in obese and overweight T2DM patients:

Serum haptoglobin levels of both wild and heterozygous PPARγ2 polymorphic obese and overweight T2DM subjects remained within the normal levels (Table 3).

## Frequency of the PPAR gamma 2 wild and heterozygous alleles in obese and overweight South Indian T2DM patients:

Our study with 180 obese and overweight South Indian T2DM patients indicated that about 92.2% of overweight and 62.2% of obese T2DM patients had wild (CC) PPAR-γ 2 polymorphism while heterozygous (CG) PPAR-γ polymorphism was observed in 7.78% of overweight and 37.78% of obese T2DM patients (Table 1).

##  Lipid profiles of obese and overweight T2DM patients with PPAR gamma 2 polymorphism:

Lipid profiles analysed for both wild and heterozygous PPARγ2 polymorphic obese and overweight T2DM subjects demonstrated an elevation in triglycerides, LDL, and VLDL (Table 2) indicating an increased risk of macro vascular complications [12].

## Role of the atherogenic index and its association with PPAR gamma 2 polymorphism in obese and overweight T2DM patients:

**Figure 4:**
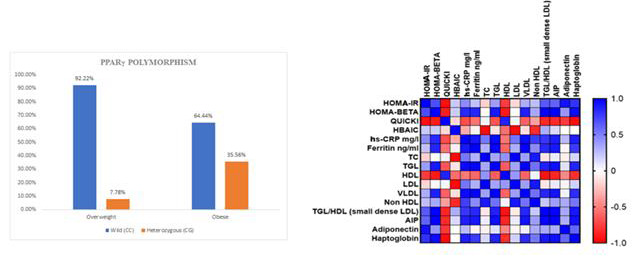
Comparative analysis of PPARγ with respect to polymorphism type in both obese and overweight and differential regulation of various biomedical parameters.

**Figure 5:**
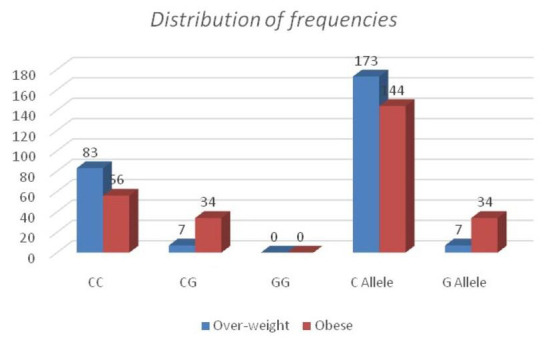
Distribution of frequencies of PPARG (PRO12 ALA) gene

The atherogenic index of plasma was computed on the basis of HDL and TAG. The index indicates the increased risk and susceptibility to cardiovascular disease in both wild and heterozygous PPARγ 2 polymorphic obese and overweight T2DM patients (Table 2). Figure 4 depicts the %PPARγ 2 wild type polymorphism and %PPARγ heterozygous polymorphism observed in obese & overweight T2DM patients; Figure 5 illustrate the levels of biochemical parameters HOMA-IR, HOMA-BETA, HBA1C, QUICKI, Hs-CRP, Haptoglobin, Adiponectin in wild type and heterozygous PPARγ 2 polymorphic obese and overweight T2DM patients; indicates the lipid profile of obese & overweight T2DM patients.

Table 4 conveys the result that Ferritin behaves differently with and without reference to the gene polymorphism. Gene allele frequencies were calculated by gene counting method.In order to compute the expected proportions under Hardy Weinberg, the allele frequencies of that particular gene were estimated. The method used, counting the number of each type of allele in the sample and dividing by the total number of alleles in the sample is referred to as gene counting [13].The study observations for overweight and obese group's observations were checked for the deviation from the Hardy-Weinberg equilibrium. Allele and genotype distribution among groups were evaluated using chi-square test. The difference in frequencies between the over-weight and obese groups were analyzed for statistical significance at 95% Confidence Interval (CI) using Chi-square test. All analyses were done by using IBM SPSS Statistics version 20 (SPSS Inc., Chicago, USA). A p-value more than 0.05 was considered as non-significant and p-value less than 0.05 was considered as significant.

## GENETIC-SNP analysis:

The frequencies of the distributions of PPARG (Pro12 Ala) genotypes in the two study groups were counted by gene counting method after confirmation of the respective allele by the gel electrophoresis.

The observational results of the frequencies of the alleles of PPARG (PRO12 ALA) gene are as shown below

The allelic frequencies obtained were subjected to analysis for Hardy-Weinberg law of genetic equilibrium. Upon analysis, it has been found that the observations were in accordance with Hardy-Weinberg law of genetic equilibrium and that the frequencies obtained in the study represent the population.

From the observation in the above table 12, it can be found that the frequencies of PPARG (Pro12 Ala) in overweight group the *p*-value obtained is more than 0.05 while in obese group the *p*-value obtained is less than 0.05. It can be concluded that the allelic frequencies are in accordance with Hardy-Weinberg law in over-weight group and it can also be further stated that they represent the population. The results obtained were found to be in Hardy-Weinberg equilibrium Table 5,Table 6.

## PPARG (Pro 12 Ala) gene SNPs:

After confirming that the frequencies are in accordance with Hardy-Weinberg Equilibrium, the frequencies are further statistically analysed using the 2 x 2 chi-square test and odds-ratio analysis.

The results indicated in Table 7 clearly denote that the minor allele G containing heterozygous CG, as exemplified therein is around 8 and 38 percent respectively in overweight and obese.

Upon analysis of PPARG (Pro12 Ala) gene SNP, it is found that the occurrence of C-allele was 96.11% in over-weight group whereas 81.11% in obese group. Further, G-allele was 3.89% in overweight group and 18.89% in obese group (Figure 5).Upon analysis of the homozygous wild alleles CC and heterozygous alleles CG, the occurrence of the CG polymorphisms of PPARG (Pro12 Ala) was significant (OR 0.1389, (0.0575 to 0.3353); with *p* < 0.0001).It can be stated that the occurrence of heterozygous alleles was statistically significant as shown in table-7.

## Discussion:

PPAR- γ is known to be a significant player in controlling the process of adipogenesis. With respect to the expression pattern of the PPAR-γ, it is found in abundance in adipose tissue, though an average amount of PPAR-γ mRNA is reported in other organs including skeletal muscle, colon, and especially lung [14]. It possesses 2 isoforms, and PPARγ1 it is known to be expressed in non-adipose tissues. The second one called as PPAR-γ2 which is specific to adipose tissues is linked to insulin resistance.Pro12Ala polymorphism is widely believed to be linked to increased insulin sensitivity. This action is attributed to several factors including decreased release of free fatty acids from the adipose tissue, where the isoform PPAR gamma 2 is exclusively expressed. Hence, it is imperative to understand that the modulation of expression and release of adipocytokines that influence insulin sensitivity are significant mechanisms portraying the role of PPAR gamma 2 in T2DM.PPAR gamma is a key regulator of the nodal relationships involving a wide gamut of facets that include nutrients, gullibility to obesity, and control of molecules released from adipocytes, and most significantly insulin sensitivity. Evidences are available to cite the fact that the alanine allele of the Pro12Ala polymorphisms in the PPAR gamma 2 is associated with reduced risk for T2DM and also the progression of insulin resistance. BMI is an important factor considering the diverse effects of Pro12Ala polymorphism on the T2DM risk. Asians with the Ala12 allele (35%) have a lower risk than Europeans and Northern Americans with the Ala12 genotype compared to their Pro12 allele controls [15]. If this difference is adjusted for the BMI of controls between Asians and Europeans then it is not significant. These suggest that the Ala12 allele also has a role in several populations with lower BMI in preventing the pathogenesis of T2DM.Dietary lipid levels are also influencing factors for the defensive role of the Ala12 allele against T2DM. A study on 305 Egyptian patients had concluded that PPAR-γ Pro12Ala polymorphism correlates with obesity and other metabolic syndrome factors in diabetic patients with cardiovascular complications [16]. But in the case of the Indian population, this polymorphism is differently reported. β cell dysfunction, increased hs-CRP level, increased triglycerides, LDL, VLDL, and AIP are interrelated. PPAR γ 2 polymorphism is known to contribute significantly to the pathological process of T2DM [17]. In this study, we find that both wild and heterozygous PPARγ2 polymorphic obese and overweight T2DMhave a different magnitude of insulin resistance. Similarly, when the beta cell function was analyzed in both wild and heterozygous PPAR-γ2 polymorphic obese and overweight T2DM a significant change was perceptible (HOMA-BETA). The inflammatory biomarker hs-CRP is strongly linked with insulin resistance in obese T2DM patients [18]. Abnormal lipid profiles were observed in all four groups (wild obese, wild overweight, heterozygous obese, heterozygous overweight T2DM). Pearson's correlation matrix depicts a significant correlation (>8) between insulin resistance, TC, and LDL. Studies by Zheng *et al*., suggest that dyslipidemia in individuals with impaired glucose tolerance and insulin resistance could result in further deterioration of the βcell function [19]. Serum adiponectin levels were also found to have a significant decline in the four groups validating its negative correlation with insulin resistance and dyslipidemia. Furthermore, some studies indicate that elevated AIP in T2DM patients could increase cardiovascular disease risk with poor prognosis[20].The present study is unique in the sense that we had attempted to project a relationship involving insulin resistance, inflammatory mediators (hs-CRP, haptoglobin, Ferritin and adiponectin), with reference to the wild type and heterozygous variants of PPARγ gene. The relationship was elicited in anthropometrically distinct male diabetics (overweight and obese). The study opens newer vistas in the frontiers of personalized medicine concerning therapeutic modalities for type 2 diabetes mellitus (overweight and obese males).

## Conclusion:

The wild type and heterozygous variant of PPARγ2 depicted significance with the inflammatory mediator, namely haptoglobin, whereas adiponectin, an adipokine exhibited significance in the wild type (obese vs non-obese type 2 diabetics). Chi-square test was performed to assess the relation between polymorphic genotypes and ferritin emerged significant. Indices of insulin sensitivity/ insulin resistance depicted different characteristics with wild type and heterozygous variant of PPARγ2 gene. The inflammatory mediators (hs-CRP, Ferritin, Haptoglobin and Adiponectin) exhibited variegated characteristics with the wild type and heterozygous variant of PPARγ2, thus pointing to the nexus among insulin resistance, inflammation, and adipocyte differentiation. However, the nexus clearly demarcated the obese from non-obese type 2 diabetics. The present study is an attempt aimed at providing newer insights into personalized medicine for enabling the treatment of patients with type 2 diabetes mellitus

## Figures and Tables

**Table 1:** Distribution of PPAR gamma 2 polymorphism-Overweight vs. obese.

**SNP**	**Genotype**	**Over-weight(n=90)**	**Obese (n=90)**	**Odds ratio 95 % CI**	**z statistic**	**Significance level**
PPARγ2SNP (Pro12Ala)	CC	83(92.22%)	56(62.22)	0.1389 (0.0575 to 0.3353)	4.39 0	P < 0.0001
	CG	07(7.78%)	34(37.78)	0.6766 (0.0132 to 34.6019)	0.195	P = 0.8457
	GG	00(0%)	00(0%)			
	C Allele	173(96.11%)	146(81.11%)	0.1738 (0.0748 to 0.4036 )	4.07	P <0.0001
	G Allele	07(3.89%)	34(18.82%)			
The results indicated in Table 1 clearly denote that the heterozygous CG, as exemplified therein is around 8 and 38 percent respectively in overweight and obese respectively.

**Table 2:** Genotyping of PPAR gamma 2 with reference to anthropometry specified parameters (obese vs. Overweight) as a function of biochemical parameters (independent of lipid profile) including insulin sensitivity and insulin resistance

**Parameters**	**Heterozygous (CG)**			**Wild type (CC)**		
	Obese (n = 32)	Overweight (n = 7)	P value	Obese (n = 58)	Overweight (n = 83)	P value
	(Mean ± SD)	(Mean ± SD)		(Mean ± SD)	(Mean ± SD)	
HOMA IR	4.716 ± 2.362	3.087 ± 0.7427	0.0818	6.022 ± 6.607	3.834 ± 2.768	0.0078
HOMA BETA	68.23 ± 81.64	24.47 ± 11.98	0.0108	51.78 ± 42.03	37.27 ± 44.92	0.0547
QUICKI	0.3106 ± 0.019	0.324 ± 0.012	0.0885	0.3088 ± 0.026	0.3204 ± 0.019	0.003
HBA1C	9.109 ± 2.394	7.814 ± 1.276	0.1761	9.286 ± 2.026	10.83 ± 12.99	0.372
TC	204.7 ± 26.28	207 ± 4.761	0.822	201.5 ± 28.29	196.3 ± 34.22	0.345
TGL	196.2 ± 52.18	179.4 ± 33.88	0.423	183.4 ± 44.42	178.7 ± 45.80	0.544
HDL	40.22 ± 7.019	43.57 ± 4.504	0.236	40.67 ± 5.841	41.27 ± 7.107	0.601
LDL	125.3 ± 24.04	127.4 ± 10.39	0.824	122.3 ± 31.20	117.1 ± 34.35	0.354
VLDL	41.22 ± 14.72	36 ± 6.782	0.368	36.88 ± 9.272	35.66 ± 9.119	0.44
Non-HDL	164.5 ± 24.56	163.4 ± 5.563	0.9127	162.6 ± 29.62	151.9 ± 32.48	0.0487
TGL/HDL (small dense LDL)	4.924 ± 1.268	4.057 ± 0.509	0.686	4.575 ± 1.118	4.404 ± 1.473	0.457
AIP	0.3136 ± 0.111	0.2504 ± 0.050	0.154	0.2833 ± 0.105	0.265 ± 0.128	0.384
p value < 0.05* (significant); P value < 0.001# (highly significant)

**Table 3:** Genotyping of PPAR gamma 2 with reference to anthropometry specified parameters (Obese vs. Overweight) as a function of inflammatory mediators

**Parameters**	**Heterozygous**			**Wild type**		
	Obese (n = 32)	Overweight (n = 7)	P value	Obese (n = 58)	Overweight (n = 83)	P value
	(Mean ± SD)	(Mean ± SD)		(Mean ± SD)	(Mean ± SD)	
hs-CRP mg/l	5.481 ± 3.468	4.200 ± 2.831	0.368	4.584 ± 3.301	3.735 ± 3.238	0.13
Ferritin ng/ml	230.9 ± 188.8	86.9 ± 53.45	0.055	101.4 ± 69.77	91.07 ± 66.68	0.3748
Adiponectin	0.816 ± 0.509	0.372 ± 0.189	0.03	2.485 ± 12.52	0.387 ± 0.253	0.128
Haptoglobin	1.573 ± 0.695	0.245 ± 0.047	0.0001	1.294 ± 0.739	0.355 ± 0.284	0.0001

**Table 4:** Chi-square analysis for the selected candidate

**Chi-square analysis - Variables in the Equation**							
		**B**	**S.E.**	**Wald**	**df**	**Sig.**	**Exp(B)**
Step 1^a^	Ferritin ng/ml	-0.005	0.002	8.858	1	0.003	0.995
	hs-CRP mg/l	-0.084	0.048	3	1	0.083	0.92
	Adiponectin	0.697	0.884	0.622	1	0.43	2.009
	haptoglobin	-0.471	0.774	0.37	1	0.543	0.624
	Constant	0.851	0.378	5.061	1	0.024	2.341
a. Variable(s) entered on step 1: Ferritin ng/ml, hs-CRP mg/l, Adiponectin, haptoglobin.							

**Table 5:** Frequency observations of PPARG (Pro12 Ala) gene alleles

**S.No.**	**Gene**	**allele**	**Frequencies**	
			Overweight Group	Obese Group
1	PPARG (PRO12 ALA)	CC	83	56
		CG	7	34
		GG	0	0
	Total		90	90

**Table 6:** Hardy-Weinberg Equilibrium Test of PPARG (Pro12 Ala) gene

**Gene**	allele	**Frequencies**	
		Over-weight Group	Obese Group
PPARG (Pro12 Ala)	CC	83	56
	CG	7	34
	GG	0	0
Hardy-Weinberg Equilibrium Test (Chi-Square Test)		*p-value* = 0.7011	*p-value* = 0.0371

**Table 7:** Distribution of polymorphisms in PPARG (Pro12 Ala) gene in over-weight and obese groups

**SNP**	**Genotype**	**Over-weight (n=90)**	**Obese (n=90)**	**Odds ratio 95 % CI**	**z statistic**	**Significance level**
PPARG (Pro12 Ala) gene	CC	83(92.22%)	56(62.22)	0.1389(0.0575 to 0.3353)	4.39	P < 0.0001
	CG	07(7.78%)	34(37.78)	0.6766 (0.0132 to 34.6019)	0.195	P = 0.8457
	GG	00(0%)	00(0%)			
	C Allele	173(96.11%)	146(81.11%)	0.1738 (0.0748 to 0.4036 )	4.07	
	G Allele	07 (3.89%)	32 (18.89%)			P <0.0001
